# Subclinical Changes in Deceased Donor Kidney Proteomes Are Associated With 12-month Allograft Function Posttransplantation—A Preliminary Study

**DOI:** 10.1097/TP.0000000000002358

**Published:** 2019-01-28

**Authors:** Maria Kaisar, Leon van Dullemen, Philip Charles, Zeeshan M. Akhtar, Marie L. Thézénas, Honglei Huang, Astrid Klooster, Nicholas A. Watkins, Benedikt Kessler, Rutger J. Ploeg

**Affiliations:** 1 Nuffield Department of Surgical Sciences, University of Oxford, Oxford, United Kingdom.; 2 Target Discovery Institute, Nuffield Department of Medicine, University of Oxford, Oxford, United Kingdom.; 3 Research and Development, NHS Blood and Transplant, Bristol, United Kingdom.; 4 Surgical Research Laboratory, University of Groningen, Groningen, The Netherlands.; 5 Oxford Biomedical Research Centre, Oxford, United Kingdom.; 6 Pathology Department, Pathologie Friesland, Leeuwarden, The Netherlands.

## Abstract

**Background:**

Cerebral injury during donation after brain death may induce systemic damage affecting long-term kidney function posttransplantation. Conventional evaluation of donor organ quality as a triage for transplantation is of limited utility.

**Methods:**

We compared donor kidneys yielding opposing extremes of the continuum of posttransplantation outcomes by several common kidney biopsy evaluation techniques, including Kidney Donor Profile Index and Remuzzi scoring, and analyzed tissue from a minimal sample cohort using label-free quantitation mass spectrometry. Further assessment of the proteomic results was performed by orthogonal quantitative comparisons of selected key proteins by immunoblotting.

**Results:**

We show that common evaluation techniques of kidney biopsies were not predictive for posttransplantation outcomes. In contrast, despite the limited cohort size, the proteomic analysis was able to clearly differentiate between kidneys yielding extreme posttransplantation outcome differences. Pathway analysis of the proteomic data suggested that outcome-related variance in protein abundance associated with profibrotic, apoptosis, and antioxidant proteins. Immunoblotting confirmation further supported this observation.

**Conclusions:**

We present preliminary data indicating that there is scope for existing evaluation approaches to be supplemented by the analysis of proteomic differences. Furthermore, the observed outcome-related variance in a limited cohort was supported by immunoblotting and is consistent with mechanisms previously implicated in the development of injury and cytoprotection in kidney transplantation.

## Abstract 2

Proteomic analyses using mass spectrometry and immunoblot of a small cohort of kidneys from brain death donors is superior to predict graft function (estimated glomerular filtration rate at 3 and 12 months) versus clinical and histological standard classifications. Supplemental digital content is available in the text.

Organ transplantation is a life-saving and life-transforming treatment of patients with end-stage organ disease. There is a persistent shortage of deceased donor organs; in the United Kingdom, between April 2016 and March 2017, 457 patients on the waiting list died and 875 became too ill to receive a transplant.^1^ Because of an aging population with a higher incidence of comorbid conditions, the demand for transplants may further increase as a consequence of increasing prevalence of diabetes, hypertension, and obesity. In response, the deceased donor pool has been expanded to include higher-risk donors who are older or have comorbidities.^2-4^ Donors older than 60 years now represent almost 40% of the donation after brain death (DBD) donors in the United Kingdom.^5^ Kidneys obtained from this “extended criteria” category have suboptimal transplant outcomes when compared with those from “standard criteria” DBD or living donors.^6,7^

The decision to use or reject a donor organ is made at the clinical level based on an evaluation of organ quality. Current methods of kidney assessment combine surrogate markers of kidney function during donor management, known risk factors such as donor age, and histological evaluation. These assessments have a high degree of subjectivity and are imperfect predictors of posttransplantation organ performance. Clinicians are thus conservative in accepting organs from “high-risk” donors. In consequence, many useable kidneys may be declined as transplants.^8,9^ Improving the discriminatory power of the diagnostic tools available to clinicians could help increase deceased donor organ utilization. This is a major goal of the UK Quality in Organ Donation (QUOD) biobank, a recently established nationwide project to collect longitudinal blood, urine, and biopsy samples from deceased donors and link these samples to demographic and clinical data for both donor and recipient.^10^

The evolution and application of mass spectrometry (MS) techniques in medical research, including in the field of transplantation, has allowed for monitoring and near simultaneous analysis of thousands of proteins and has led to clinically relevant findings.^11-14^ In this study, we applied proteomic analysis to a minimal cohort of QUOD biobank samples to investigate whether is feasible to identify a proteomic profile of donor kidneys that may add predictive value to current assessment methods of donor kidney quality with regard to posttransplantation outcome, which would justify a subsequent large-cohort study using many tissue samples. To this end, we compared the kidney proteomes of donor groups with clearly defined extreme posttransplantation outcomes (suboptimal vs good), matching as many clinical and demographic parameters as possible, to assess whether observable proteomic differences were able to discriminate between outcomes. The observed differences in this limited-cohort comparison were verified for the biological relevance of implicated pathways by selective immunoblotting.

## MATERIALS AND METHODS

### Study Population

The QUOD biobank is a national bioresource that houses an extensive repository of samples from deceased donors obtained at specific time points according to predefined collection protocols during donor management and organ procurement throughout the United Kingdom. Donor samples are linked to corresponding donor and recipient demographic and clinical data. Appropriate informed consent by the donor families precedes sample collection. All kidney biopsy samples analyzed in this study were procured from donors after brain death and obtained from the UK QUOD biobank, under the ethical approval of the QUOD project 13/NW/0017. Kidney biopsies were obtained ex situ from the upper pole of kidney cortex during preparation at the back table using a 23 mm needle biopsy gun. Each biopsy specimen was divided in two; one half was stored in RNAlater followed by subsequent storage in liquid nitrogen and the other half in formalin.

### Clinical Variables

Estimated glomerular filtration rate (eGFR) was calculated using the four-variable Modification of Diet in Renal Disease formula and is expressed in milliliters per minute, adjusted for body surface area.^15^ The Kidney Donor Profile Index (KDPI) was calculated using donor age, height, weight, serum creatinine at retrieval, hepatitis C virus status, history of hypertension, diabetes, cause of death and Donation after Circulatory Death category using an online calculator^16^ (References 16-45 can be found in **Material and Methods, SDC,** http://links.lww.com/TP/B599; References section). The incidence of acute kidney injury in the donors was also assessed using Acute Kidney Injury Network (AKIN) scoring, by calculating the fold change between the levels of terminal and the baseline serum creatinine at donor admission in intensive care^17^. A fold change of less than 1.5 was defined as no AKIN, a fold change of 1.5 to 2 as AKIN-1, a fold change of 2 to 3 as AKIN-2, and a fold change of more than 3 as AKIN-3.

### Selection of Sample Cohort

Biopsy samples were selected from donors on the basis of the 3-month posttransplantation outcomes of the pairs of kidney recipients from the same donor (**Figure S1A, SDC**, http://links.lww.com/TP/B599). To reduce the impact of recipient-related variation on outcomes, only instances where both kidneys from a donor yielded similar transplant outcomes were considered. On this basis, two experimental groups were formed; suboptimal (SO) and good outcome (GO) (**Figure S1A, SDC**, http://links.lww.com/TP/B599). To select the eligible donors per group, we set an upper eGFR limit for SO (eGFR ≤39 mL/min/1.73 m^2^) and lower eGFR limit for GO (eGFR ≥ 50 mL/min per 1.73 m^2^) using data from the UK Transplant Registry, published yearly, as a guide.^18^ Good outcome donor kidneys functioned immediately after transplantation and had a mean 3-month eGFR of 65.2 ± 8 mL/min per 1.73 m^2^ (25th and 75th percentiles for eGFR were 62 and 70.25 mL/min per 1.73 m^2^, respectively). The SO donor kidneys developed delayed graft function (a need for dialysis during the first week posttransplant excluding urinary tract obstruction, hyperkalemia or fluid overload) after transplantation and had a mean 3-month eGFR of 29.8 ± 7 mL/min per 1.73 m^2^ (25th and 75th percentiles for eGFR were 37.2 and 24.8 mL/min per 1.73 m^2^, respectively).

The difference of the posttransplant 3-month eGFR values between the two cohorts (SO vs GO) was significant different (*P* < 0.0001; Mann-Whitney test) (Table 1).

**TABLE 1:**
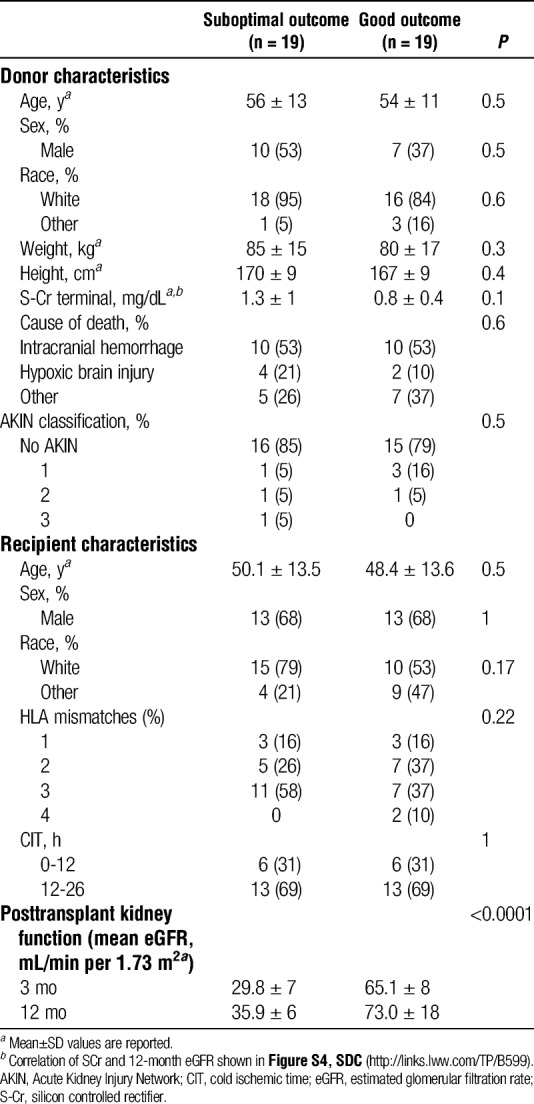
Donor and recipient demographic and clinical characteristics associated with the analyzed kidney samples

Donors and recipients were further matched for demographic and clinical characteristics to minimize biological heterogeneity (Table 1). Only donor kidneys with completed posttransplant longitudinal clinical data for both recipients were included in the analysis. Biopsy samples from one kidney per donor were included in the study. Biopsies from 38 donors, 19 donors with SO and 19 donors with GO, were analyzed.

### Histopathological Assessment

Baseline donor chronic kidney injury was assessed retrospectively using the formalin stored, paraffin-embedded part of the core needle biopsy. Biopsies were evaluated by an experienced histopathologist (blinded to the donor characteristics) and scored for chronic histopathological changes according to the classification system introduced by Remuzzi and colleagues.^19^ Kidney paraffin sections (4 μm) were dewaxed, rehydrated, and stained with 0.5% periodic acid for 5 minutes, rinsed with distilled water, and then placed in Schiff reagent for 15 minutes followed by 5 minutes washing in tap water. The slides were counterstained with Mayer hematoxylin and washed in tap water for 5 minutes.

### Selection of a Minimal Sample Cohort for Proteomic Analysis

A subgroup of donor samples was randomly selected from the overall cohort of 38 donor kidney biopsies for proteomic analysis (**Figure S1B, SDC**, http://links.lww.com/TP/B599). Sample size was selected such that relatively substantial changes (twofold or more) were predicted be measured reliably (setting power at 0.8 and confidence threshold at 0.05), assuming a combined technical and biological variation of 50%, whereas minimizing the number of biological replicates and thus tissue sample usage. The selected minimum cohort subset size of five per condition was informed by previously published estimation of experimental power in quantitative proteomics.^20^ The rest of the donor samples (n = 14 SO; n = 14 GO) were used for immunoblotting analysis (**Figure S1B, SDC**, http://links.lww.com/TP/B599) that was performed to verify observed outcome-related variance between the two groups SO versus GO (**Materials and Methods, SDC**, http://links.lww.com/TP/B599; immunoblotting analysis).

### Proteomic Analysis by MS

Kidney cortical biopsy samples from 10 donors were lysed in 300 μL of RIPA buffer (150 mM NaCl, 1.0% NP-40, 0.5% sodium deoxycholate, 1% SDS, 50 mM Tris, pH 8.0) containing protease (Roche, USA) and phosphatase inhibitor cocktails (Sigma, UK). Homogenization was performed on a bead beater at 6500 rpm for 3 cycles of 40 seconds each with intermediate 1 minute on ice between cycles. Label-free quantitation MS analysis was performed as previously described.^21^ In brief, 15 μg total protein material per sample was reduced for 1 hour by addition of 200 mM dithiothreitol (DTT) followed by alkylation with 200 mM iodoacetamide for 30 minutes at room temperature. Trypsin digestion was performed overnight at 37°C with gentle mixing using a 1:50 trypsin:protein ratio. Samples were acidified with 1% formic acid or trifluoroacetic acid. Peptide digests were then desalted using Sep-Pak C18 cartridges (Waters) and dried by Speed Vac centrifugation. Pellets were re suspended in 30 μL of buffer A (98% Milli-Q-H_2_O, 2% acetonitrile, 0.1% formic acid) before MS analysis. Peptides were analyzed in duplicates using a C18 column (75 μm × 250 mm, 1.7 μm particle size)on a Dionex Ultimate 3000 nano-ultrahigh-performance liquid chromatography system (Thermo Scientific, Bremen, US) coupled to a Q Exactive mass spectrometer (Thermo Scientific, Bremen, Germany). The MS data were processed and identified proteins quantified using the Central Proteomics Facilities Pipeline.^22^ Normalized Spectral INdex Quantitation values, were calculated for each protein.^23^

### Statistical Analysis

Differences in demographic and clinical characteristics of donors and their corresponding kidney recipients between SO and GO groups were examined by Mann-Whitney test for continuous variables (mean ± standard deviation [SD]) and by χ^2^ or Fisher exact test for discrete variables and to define whether the association of donor organs to the transplantation outcomes is independent of the listed variables as listed in Table 1.

A nonsupervised analysis of proteomic changes by Principle Component Analysis was performed and the first two principle component dimensions visualized using R (v3.4.2). Proteins with differential levels of abundance between the nonmissing values in the two subgroups (*P* < = 0.05, *t* test, no multiple-testing correction) and identification in at least 3 of 5 analyzed samples per group were further analyzed in a supervised manner using hierarchical clustering using the PermutMatrix software.^24^ Missing abundance values were imputed for each protein by the mean abundance of that protein within each condition. Dissimilarity between the donors was assessed by single linkage (closest neighbor linkage) for columns and rows.

## RESULTS

We formed two cohorts of demographically and clinically matched donor kidneys, where the donor had offered both kidneys as single transplants that had similar outcomes, based on SO versus GO short- and long-term allograft functions. We initially selected donors on the basis of 3-month eGFR, and subsequently, we acquired the 12-month posttransplantation eGFR values. Recipients in the SO group had mean 3-month eGFR (±SD) 29.8 ± 7 ml/min/1.73 m^2^ and 12-month eGFR 35.9 ± 6 mL/min per 1.73 m^2^, whereas recipients within the GO group had mean 3-month eGFR 65.1 ± 8 mL/min per 1.73 m^2^ and 12-month eGFR 73 ± 18 mL/min per 1.73 m^2^.

**FIGURE 1:**
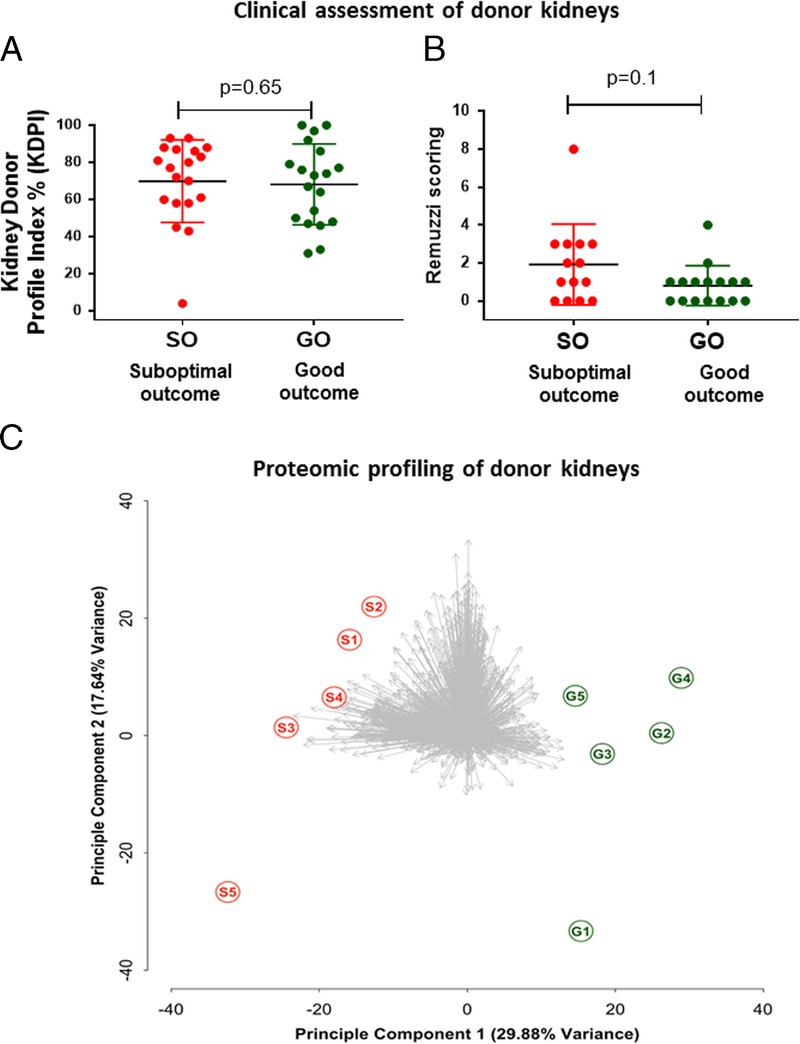
Clinical assessment of donor kidney biopsies by Kidney Donor Profile Index (KDPI) and Remuzzi scoring and proteomic profiling. A, KDPI and Remuzzi scoring failed to discriminate the donor kidneys in risk of suboptimal transplantation outcomes. KDPI scoring was performed using the KDPI online calculator; there was no significant difference between the KDPI scoring of the donors included in the study (Mann-Whitney test; *P* = 0.69; data show mean ± standard deviation [SD]). B, Histological analysis shows there was no significant difference between the Remuzzi scoring of the donors included in the study (Mann-Whitney test; *P* = 0.1; data show mean ± SD). C, Proteomic profiling of donor kidneys. Kidney tissue proteome profiles discriminate donors by unsupervised principle component analysis on the basis of allograft function after transplantation.

Kidney donor profile index evaluation showed no association between KDPI score and subsequent SO or GO assignment (*P* = 0.65, Mann-Whitney test). The 25th percentile KDPI values were 58% and 47.7% for SO and GO, respectively, while the 75th percentile KDPI values were identical for both experimental cohorts at 87.5% (Figure 1A). Evaluation of the donor biopsies for chronic kidney disease by Remuzzi scoring was performed on biopsies from 30 of the donors; biopsies from the remaining donors had insufficient number of glomeruli. Sixty-eight percent of the donor kidneys with SO and 79% of donor kidneys with GO had a Remuzzi score of 0 to 3, which did not yield a significant association with outcome (*P* = 0.1, Mann Whitney test) (Figure 1B and **Table S1, SDC**, http://links.lww.com/TP/B599). The low *P* value does suggest that the Remuzzi comparison lacked power, resulting in a false negative, although this would still imply that the scoring system is insufficiently sensitive. Assessing the onset of acute kidney injury during donor management using AKIN classification revealed that the majority of donors did not show indications of acute kidney injury with 85% of the donor kidneys with SO and 74% with GO classified as no AKIN; again, these results did not associate with outcome (*P* = 0.5, Mann-Whitney *U* test, Table 1).

We selected a minimal subset (n = 5 SO vs n = 5 GO) from the full cohort for proteomic profiling of major fold changes (FC > =2, *P* < = 0.05, power = 0.8). We identified and quantified 1743 unique proteins at a false discovery rate (FDR) of <=1%. Unsupervised principle component analysis of this data set demonstrated that the two groups were clearly resolved by the first principle component (representing a plurality of 29.88% of the total variance in the data set), demonstrating substantial merit for prediction of transplant outcome by proteomic methods (Figure 1C).

**FIGURE 2:**
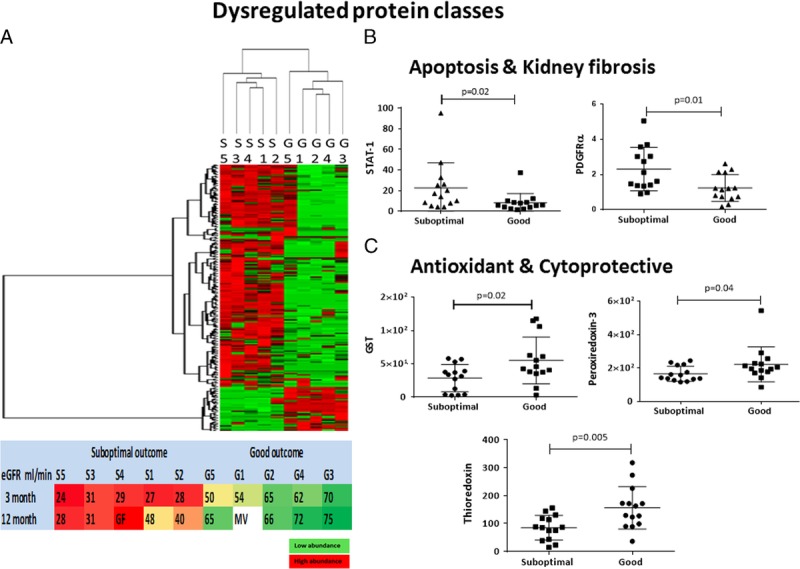
Dysregulated protein classes in kidney tissue between donors with suboptimal and good transplantation outcomes. A, Supervised hierarchical cluster analysis segregated individual donor kidneys in two distinct groups according to 3-month kidney function posttransplantation. S1, S2, S3, S4, S5: Individual donor kidneys with suboptimal (SO) G1, G2, G3, G4, G5: Individual donor kidneys with good outcome (GO) Association between the hierarchical cluster analysis–derived dendrogram from donor kidney biopsies analysis and the recipient kidney function recorded at 3-month (used for selection) and 12-month posttransplantation (retrospective). GF, graft failure; MV, missing value. B, STAT-1 and PDGFRα are enriched in donor kidneys with SO. Western blot analysis of STAT-1 and PDGF Rα on the rest of the selected sample cohort of n = 28 biopsy samples (n = 14 suboptimal and n = 14 GO cohort). Normalized by ß-actin, densitometry analysis shows significant increased levels of STAT-1 and PDGFRα in SO associated donor kidney biopsies (*P* ≤ 0.05; data show mean ± standard deviation [SD]). C, Cytoprotective proteins are enriched in the donor kidneys with GO. Western blot analysis of GST, PRX3 and TRX1 on the rest of the selected sample cohort n = 28 donation after brain death kidney biopsies (n = 14 suboptimal and n = 14 GO cohort). Normalized by ß-actin densitometric analysis shows significant increased levels of TRX1, GST, and PRX3 in GO associated donor kidney biopsies (*P* ≤ 0.05; data show mean ± SD).

We next performed a supervised clustering analysis on a subset of proteins showing potential differences between the two groups (SO vs GO). Thresholding by *t* test (*P* value ≤0.05) and presence in at least 3 of 5 samples per group gave a subset of 214 proteins. Hierarchical clustering analysis using this subset easily segregated the donor kidneys by transplant outcome. Comparing this clustering to long-term outcome as measured by 12-month eGFR illustrated the close association between the proteins with significant differences and long-term kidney function (Figure 2A).

We examined the set of proteins with significant statistical differences between the two outcomes to verify the overarching trends. Pathway analysis via STRING^25^ of this 214-protein subset suggested enrichment of cellular metabolic processes that included cellular response to stress (n = 26 proteins; FDR: 2 × 10E−3) and cell surface receptor signaling pathways (n = 40; FDR: 3 × 10E−3) in SO versus GO, and enrichment of reactive oxygen species (ROS) detoxification (n = 107 proteins; FDR: 2 × 10E−3) in GO versus SO. Among the predominant pathways (via Kyoto Encyclopedia of Genes and Genomes^26^ database) were metabolic dysregulation (n = 107, FDR: 7.13 × 10E−6) and tight junction molecules (n = 40 proteins; FDR: 3 × 10E−3).

As a further assessment of the proteomic results, we performed orthogonal quantitative comparisons of selected key proteins by immunoblotting (**Figure S1B, SDC**, http://links.lww.com/TP/B599). In SO versus GO, we were able to reproduce the observation of significantly increased expression of the apoptotic signal transduction protein STAT-1, observed in proteomics data with sevenfold SO/GO (*P* = 0.03) and by immunoblot with *P* = 0.02 (Figure 2B and **Figure S2A, SDC**, http://links.lww.com/TP/B599). The proteomics data also suggested a role for a profibrotic response, with TGF-β1 (observed in proteomics data with sevenfold SO/GO, *P* = 0.034). We were unable to quantify TGF-β1 confidently due to technical noise; however, immunoblotting of PDGFRα (which has a synergetic role in the onset and propagation of fibrosis) did indicate elevation (*P* = 0.01) (Figure 2B and **Figure S2B, SDC**, http://links.lww.com/TP/B599). In GO versus SO, we were able to observe enrichment of several notable antioxidant and cytoprotective proteins by Western blot, including thioredoxin-1 (*P* = 0.005), glutathione S-transferase (*P* = 0.02), and peroxiredoxin-3 (*P* = 0.04) (Figure 2C and **Figure S3A, B, C, SDC**, http://links.lww.com/TP/B599). Interestingly, catalase was not observed to be enriched by either the proteomic analysis (*P* > 0.05) or immunoblotting (*P* > 0.05) (**Figure S3D, SDC**, http://links.lww.com/TP/B599).

## DISCUSSION

Assessment of donor organ quality using current clinical decision making frequently results in a conservative donor organ selection with the consequence that a large number of potentially transplantable organs are declined.^8,9^ The limitations of the current clinical methods are highlighted in our study, wherein neither AKIN, KDPI, nor Remuzzi classification predicted which donor kidneys in our cohort would have suboptimal function after transplantation, despite limiting assessment to two “extreme” posttransplant outcome groups with minimal ambiguity.

In contrast, an exploratory proteomics study indicated significant differences in the proteomes of donor kidney biopsies from the two groups allowing clear separation by nonsupervised clustering. Furthermore, the biomechanistic differences most strongly suggested by the proteomics study (particularly a role for ROS-induced injury and recovery capability) were consistent with follow-up immunoblotting and are biologically plausible in the context of previously reported studies. As we and others have previously reported, brain death leads to dysregulation of metabolic pathways and mitochondrial dysfunction that results in ROS formation, causing cellular injury in the kidney.^27^ The dynamic relationship between ROS and TGF-β has also been examined extensively in vitro and in vivo.^28–30^ The synergistic action of TGF-β and PDGFRα can promote the differentiation of pericytes and the recruitment of fibroblasts and myofibroblasts, causing irreversible changes to the extracellular matrix, promoting fibrosis and damage to renal tubules,^28,31–34^ and PDGFRα also contributes to kidney fibrosis through a maladaptive process of wound healing.^35–37^ Furthermore, in many models of fibrosis and atherosclerosis, activation of PDGF receptors leads to STAT-1 phosphorylation in a JAK1/2-dependent manner.^38–41^ Fibrosis has been closely linked to deterioration of allograft function after transplantation.^42^ The antioxidant and cytoprotective proteins found to be elevated in GO kidneys would also be consistent with ROS-induced fibrosis playing a role in posttransplant kidney dysfunction. Antioxidant cellular mechanisms are generally activated after exposure to ROS mediated stress to reinstate a healthy cellular environment,^43–45^ and the proteomic and immunoblotting data are consistent with an elevated response in these pathways being associated with better posttransplant outcome.

Our pilot study results indicate that the current assessment methods of donor kidney quality may be augmented by proteomic profiles that indicate how the balance between injury and cytoprotection is disrupted and how this is associated with long-term allograft function. Now that feasibility has been established, future work to identify a predictive protein profile can be performed in a much larger cohort with a broader continuum of outcomes.

## Supplementary Material

SUPPLEMENTARY MATERIAL
