# Supplementary material for: Subclinical Changes in Deceased Donor Kidney Proteomes Are Associated With 12-month Allograft Function Posttransplantation—A Preliminary Study
Source: Transplantation. 2019 Jan 28;103(2):323–8. doi: 10.1097/TP.0000000000002358 (PMC6365243; doi:10.1097/TP.0000000000002358)
Supplement: SUPPLEMENTARY MATERIAL [file tp-103-323-s001.docx]

**TITLE PAGE**

**Subclinical Changes in Deceased Donor Kidney Proteomes are Associated with 12-month Allograft Function Posttransplantation – a Preliminary Study**

Kaisar M^1,2,6^, van Dullemen LFA^3+^, Charles PD^2+^, Akhtar MZ^1^, Thézénas ML^2^, , Huang H^1,2,4,^ , Klooster A^3,5^, Watkins NA^6^, Kessler BM^2◊^ & Ploeg RJ^1,4◊^

*1. Nuffield Department of Surgical Sciences, University of Oxford, OX3 7LJ, UK
2. Target Discovery Institute, Nuffield Department of Medicine, University of Oxford, OX3 7FZ, UK
3. Surgical Research Laboratory, University of Groningen, 9713 GZ, The Netherlands
4. Oxford Biomedical Research Centre, OX3 7LJ, UK*

*5. Pathology Department, Pathologie Friesland, Leeuwarden, 8917 EN, The Netherlands*

*6. Research and Development, NHS Blood and Transplant, Bristol, BS34 7QH, UK*

*+ Equal contribution
◊ Equal contribution*

**Corresponding Author:** Dr. Maria Kaisar ([maria.kaisar@nds.ox.ac.uk](mailto:maria.kaisar@nds.ox.ac.uk))

Nuffield Department of Surgical Sciences,

University of Oxford

Oxford, OX3 7LJ

United Kingdom

**AUTHORSHIP PAGE**

**Authorship:** MK, BMK and RJP designed this study. MK, AZ, NW as members of the National Management Team and the QUOD Steering Committee participated in the establishment of the QUOD biobank. RJP is the Coordinator of the QUOD consortium. Clinical evaluation of samples was performed by MK. Sample preparation for proteomic study was performed by MK, MLT and HH. Mass spectrometric analysis was performed by MLT and HH. Mass spectrometric data was processed and searched by MK and MLT. Statistical analysis of proteomic quantitation was performed by MK and PDC. Immunoblotting was performed by LvD. Remuzzi scoring was performed by AK. The paper was written by MK, PDC, BMK and RJP with input from the other authors.

**Disclosure:** The authors declare no conflicts of interest

**Funding:** This work was supported by research funding from NHS Blood and Transplant Trust Fund TF031 and Oxford Transplant Foundation award to M.K., a John Fell Fund 133/075 and Wellcome Trust grant 097813/Z/11/Z to B.M.K. and COPE FP7 grant award to R.J.P.

**ABBREVIATIONS PAGE**

AKIN, Acute Kidney Injury Network; DBD, donation after brain death; eGFR, estimated glomerular filtration rate; FDR, False Discovery Rate; GO, good outcome; KDPI, Kidney Donor Profile Index; KEGG, Kyoto Encyclopaedia of Genes and Genomes; LC-MSMS, Liquid Chromatography Tandem Mass Spectrometry; LFQ, label-free quantitation; NHSBT, NHS Blood and Transplant; QUOD, Quality in Organ Donation [Biobank]; MS, Mass Spectrometry; SO, suboptimal outcome; UHPLC, ultra-high-performance liquid chromatography

**ABSTRACT**

**Background:** Cerebral injury during Donation after Brain Death (DBD) may induce systemic damage affecting long-term kidney function posttransplantation. Conventional evaluation of donor organ quality as a triage for transplantation is of limited utility.

**Methods:** We compared donor kidneys yielding opposing extremes of the continuum of posttransplantation outcomes by several common kidney biopsy evaluation techniques including Kidney Donor Profile Index (KDPI) and Remuzzi scoring, and analysed tissue from a minimal sample cohort using Label-Free Quantitation (LFQ) mass spectrometry. Further assessment of the proteomic results was performed by orthogonal quantitative comparisons of selected key proteins by immunoblotting.

**Results:** We show that common evaluation techniques of kidney biopsies were not predictive for posttransplantation outcomes. In contrast, despite the limited cohort size, the proteomic analysis was able to clearly differentiate between kidneys yielding extreme posttransplantation outcome differences. Pathway analysis of the proteomic data suggested that outcome-related variance in protein abundance associated with profibrotic, apoptosis and antioxidant proteins. Immunoblotting confirmation further supported this observation.

**Conclusions:** We present preliminary data indicating that there is scope for existing evaluation approaches to be supplemented by the analysis of proteomic differences. Furthermore, the observed outcome-related variance in a limited cohort was supported by immunoblotting and is consistent with mechanisms previously implicated in the development of injury and cytoprotection in kidney transplantation.

**MAIN BODY TEXT**

**Introduction**

Organ transplantation is a life-saving and life-transforming treatment of patients with end stage organ disease. There is a persistent shortage of deceased donor organs; in the UK between April 2016 and March 2017, 457 patients on the waiting list died and 875 became too ill to receive a transplant^1^. Due to an aging population with a higher incidence of comorbid conditions, the demand for transplants may further increase as a consequence of increasing prevalence of diabetes, hypertension, and obesity. In response, the deceased donor pool has been expanded to include higher risk donors who are older or have comorbidities^2–4^. Donors over 60 years old now represent almost 40% of the Donation after Brain Death (DBD) donors in the UK^5^. Kidneys obtained from this ‘extended criteria’ category have suboptimal transplant outcomes when compared to those from ‘standard criteria’ DBD or living donors^6,7^.

The decision to use or reject a donor organ is made at the clinical level based on an evaluation of organ quality. Current methods of kidney assessment combine surrogate markers of kidney function during donor management, known risk factors such as donor age, and histological evaluation. These assessments have a high degree of subjectivity and are imperfect predictors of posttransplantation organ performance. Clinicians are thus conservative in accepting organs from ‘high risk’ donors. In consequence, many useable kidneys may be declined as transplants^8,9^. Improving the discriminatory power of the diagnostic tools available to clinicians could help to increase deceased donor organ utilization. This is a major goal of the UK Quality in Organ Donation (QUOD) biobank, a recently established nationwide project to collect longitudinal blood, urine, and biopsy samples from deceased donors and link these samples to demographic and clinical data for both donor and recipient^10^.

The evolution and application of mass spectrometry (MS) techniques in medical research, including in the field of transplantation, has allowed for monitoring and near simultaneous analysis of thousands of proteins and has led to clinically relevant findings^11–14^. In this study, we applied proteomic analysis to a minimal cohort of QUOD biobank samples to investigate whether is feasible to identify a proteomic profile of donor kidneys that may add predictive value to current assessment methods of donor kidney quality with regard to posttransplantation outcome, which would justify a subsequent large-cohort study using many tissue samples. To this end, we compared the kidney proteomes of donor groups with clearly defined extreme posttransplantation outcomes (suboptimal versus good), matching as many clinical and demographic parameters as possible, to assess whether observable proteomic differences were able to discriminate between outcomes. The observed differences in this limited-cohort comparison were verified for the biological relevance of implicated pathways by selective immunoblotting.

**Materials and Methods**

***Study population***

The QUOD biobank is a national bio-resource that houses an extensive repository of samples from deceased donors obtained at specific time points according to predefined collection protocols during donor management and organ procurement throughout the UK. Donor samples are linked to corresponding donor and recipient demographic and clinical data. Appropriate informed consent by the donor families precedes sample collection. All Kidney biopsy samples analysed in this study were procured from donors following brain death (DBD) and obtained from the UK QUOD biobank, under the ethical approval of the QUOD project 13/NW/0017. Kidney biopsies were obtained ex situ from the upper pole of kidney cortex during preparation at the back table using a 23mm needle biopsy gun. Each biopsy specimen was divided in 2; one half was stored in RNAlater followed by subsequent storage in liquid nitrogen and the other half in formalin.

***Clinical variables***

Estimated Glomerular Filtration Rate (eGFR) was calculated using the 4 variable Modification of Diet in Renal Disease formula and is expressed in millilitres per minute, adjusted for body surface area^15^. The Kidney Donor Profile Index (KDPI) was calculated using donor age, height, weight, serum creatinine at retrieval, HCV status, history of hypertension, diabetes, cause of death and Donation after Cardiac Death category using an online calculator ^16^. The incidence of acute kidney injury in the donors was also assessed using Acute Kidney Injury Network (AKIN) scoring, by calculating the fold change between the levels of terminal and the baseline serum creatinine at donor admission in intensive care^17^. A fold change of <1.5 was defined as no AKIN, a fold change of 1.5-2 as AKIN-1, a fold change of 2-3 as AKIN-2, and a fold change of >3 as AKIN-3.

***Selection of sample cohort***

Biopsy samples were selected from donors on the basis of the 3-month posttransplantation outcomes of the pairs of kidney recipients from the same donor (Figure S1A, SDC, http://links.lww.com/TP/B599). To reduce the impact of recipient-related variation on outcomes, only instances where both kidneys from a donor yielded similar transplant outcomes were considered. On this basis, 2 experimental groups were formed; suboptimal (SO) and good outcome (GO) (Figure S1A, SDC, http://links.lww.com/TP/B599). To select the eligible donors per group, we set an upper eGFR limit for suboptimal outcome (SO: eGFR <=39ml/min/1.73 m^2^) and lower eGFR limit for good outcome (GO: eGFR >= 50ml/min/1.73 m^2^) using data from the UK Transplant Registry, published yearly, as a guide^18^ . GO donor kidneys functioned immediately after transplantation and had a mean 3-month eGFR= 65.2 +/- 8 ml/min/1.73 m^2^ (25^th^ and 75^th^ percentiles for eGFR were 62 and 70.25 ml/min/1.73 m^2^ respectively). SO donor kidneys developed delayed graft function (a need for dialysis during the first week posttransplant excluding urinary tract obstruction, hyperkalaemia or fluid overload) after transplantation and had a mean 3-month eGFR= 29.8+/-7ml/min/1.73 m^2^ (25^th^ and 75^th^ percentiles for eGFR were 37.2 and 24.8 ml/min/1.73 m^2^ respectively) .

The difference of the posttransplant 3-month eGFR values between the 2 cohorts (SO vs GO) was significant different (P<0.0001; Mann-Whitney test) (Table 1).

Donors and recipients were further matched for demographic and clinical characteristics to minimise biological heterogeneity (Table 1). Only donor kidneys with completed posttransplant longitudinal clinical data for both recipients were included in the analysis. Biopsy samples from 1 kidney per donor were included in the study. Biopsies from 38 donors, 19 donors with SO and 19 donors with GO, were analysed

***Histopathological assessment***

Baseline donor chronic kidney injury was assessed retrospectively using the formalin stored, paraffin embedded part of the core needle biopsy. Biopsies were evaluated by an experienced histopathologist (blinded to the donor characteristics) and scored for chronic histopathological changes according to the classification system introduced by Remuzzi and colleagues^19^. Kidney paraffin sections (4µm) were dewaxed, rehydrated and stained with 0.5% periodic-acid for 5 minutes, rinsed with distilled water and then placed in Schiff’s reagent for 15 minutes followed by 5 minutes washing in tap-water. The slides were counter-stained with Mayer’s haematoxylin and washed in tap water for 5 minutes.

***Selection of a minimal sample cohort for proteomic analysis***

A subgroup of donor samples was randomly selected from the overall cohort of n=38 donor kidney biopsies for proteomic analysis (Figure S1B, SDC, http://links.lww.com/TP/B599). Sample size was selected such that relatively substantial changes (2-fold or more) were predicted be measured reliably (setting power at 0.8 and confidence threshold at 0.05) assuming a combined technical and biological variation of 50%, whilst minimising the number of biological replicates and thus tissue sample usage. The selected minimum cohort subset size of 5 per condition was informed by previously published estimation of experimental power in quantitative proteomics^20^. The rest of the donor samples (n=14 SO & n=14 GO) were used for immunoblotting analysis (Figure S1B, SDC, http://links.lww.com/TP/B599) that was performed to verify observed outcome-related variance between the 2 groups SO versus GO (Materials and Methods, SDC, http://links.lww.com/TP/B599; immunoblotting analysis).

***Proteomic analysis by mass spectrometry***

Kidney cortical biopsy samples from 10 donors were lysed in 300 µL of RIPA buffer (150 mM NaCl, 1.0% NP-40, 0.5% sodium deoxycholate, 1% SDS, 50 mM Tris, pH 8.0) containing protease (Roche, USA) and phosphatase inhibitor cocktails (Sigma, UK). Homogenization was performed on a bead beater at 6500 rpm for 3 cycles of 40 seconds each with intermediate 1 min on ice between cycles. LFQ MS analysis was performed as previously described^21^. In brief, 15 µg total protein material per sample was reduced for 1 hour by addition of 200 mM dithiothreitol (DTT) followed by alkylation with 200 mM iodoacetamide (IAA) for 30 minutes at room temperature. Trypsin digestion was performed overnight at 37 °C with gentle mixing using a 1:50 trypsin:protein ratio. Samples were acidified with 1% FA or TFA. Peptide digests were then desalted using Sep-Pak C18 cartridges (Waters) and dried by Speed Vac centrifugation. Pellets were re suspended in 30 µl of buffer A (98 % Milli-Q-H2O, 2 % acetonitrile, 0.1 % formic acid) prior to MS analysis. Peptides were analysed in duplicates using a C18 column (75 μm × 250 mm, 1.7 μm particle size )on a Dionex Ultimate 3000 nano-ultra-high performance liquid chromatography system (Thermo Scientific, Bremen, US) coupled to a Q Exactive mass spectrometer (Thermo Scientiﬁc, Bremen, Germany). MS data were processed and identified proteins quantified using the Central Proteomics Facilities Pipeline^22^. Normalised Spectral INdex Quantitation (SINQ) values, were calculated for each protein ^23^.

***Statistical analysis***

Differences in demographic and clinical characteristics of donors and their corresponding kidney recipients between SO and GO groups were examined by Mann-Whitney test for continuous variables (Means+/-SD) and by Chi-squared or Fisher’s exact test for discrete variables and to define whether the association of donor organs to the transplantation outcomes is independent of the listed variables as listed in Table 1.

A non-supervised analysis of proteomic changes by Principle Component Analysis was performed and the first 2 principle component dimensions visualised using R (v3.4.2). Proteins with differential levels of abundance between the nonmissing values in the 2 subgroups (p<=0.05, *t* test, no multiple testing correction) and identification in at least 3/5 of analysed samples per group were further analysed in a supervised manner using hierarchical clustering using the PermutMatrix software ^24^. Missing abundance values were imputed for each protein by the mean abundance of that protein within each condition. Dissimilarity between the donors was assessed by single linkage (closest neighbour linkage) for columns and rows.

**Results**

We formed 2 cohorts of demographically and clinically matched donor kidneys, where the donor had offered both kidneys as single transplants that had similar outcomes, based on Suboptimal (SO) versus Good (GO) short- and long-term allograft function. We initially selected donors on the basis of 3-month eGFR and subsequently we acquired the 12-month posttransplantation eGFR values. Recipients in the SO group had mean 3-month eGFR (+/-SD) 29.8+/-7ml/min/1.73 m^2^ and 12-month eGFR 35.9+/-6 ml/min/1.73 m^2^, while recipients with in the GO group had mean 3-month eGFR 65.1+/-8ml/min/1.73 m^2^ and 12-month eGFR 73+/-18ml/min/1.73 m^2^.

Kidney donor profile index (KDPI) evaluation showed no association between KDPI score and subsequent SO or GO assignment (p=0.65, Mann-Whitney test). The 25^th^ percentile KDPI values were 58% and 47.7% for SO and GO, respectively, while the 75^th^ percentile KDPI values were identical for both experimental cohorts at 87.5% (Figure 1A). Evaluation of the donor biopsies for chronic kidney disease by Remuzzi scoring was performed on biopsies from 30 of the donors; biopsies from the remaining donors had insufficient number of glomeruli. 68% of the donor kidneys with SO and 79% of donor kidneys with GO had a Remuzzi score 0-3, which did not yield a significant association with outcome (p=0.1, Mann Whitney test) (Figure 1B & Table S1, SDC, http://links.lww.com/TP/B599). The low p value does suggest that the Remuzzi comparison lacked power, resulting in a false negative, although this would still imply that the scoring system is insufficiently sensitive. Assessing the onset of acute kidney injury during donor management using AKIN classification revealed that the majority of donors did not show indications of acute kidney injury with 85% of the donor kidneys with SO and 74% with GO classified as no AKIN; again, these results did not associate with outcome (p=0.5, Mann Whitney test , Table 1).

We selected a minimal subset (n=5 SO versus 5 GO) of the full cohort for proteomic profiling of major fold changes (FC >=2, p <= 0.05, power = 0.8). We identified and quantified 1743 unique proteins at a False Discovery Rate (FDR) of <=1%. Unsupervised principle component analysis of this dataset demonstrated that the 2 groups were clearly resolved by the first principle component (representing a plurality of 29.88% of the total variance in the dataset), demonstrating substantial merit for prediction of transplant outcome by proteomic methods (Figure 1C).

We next performed a supervised clustering analysis on a subset of proteins showing potential differences between the 2 groups (SO vs GO). Thresholding by *t* test p value <=0.05 and presence in at least 3/5 samples per group gave a subset of 214 proteins. Hierarchical clustering analysis using this subset easily segregated the donor kidneys by transplant outcome. Comparing this clustering to long-term outcome as measured by 12-month eGFR illustrated the close association between the proteins with significant differences and long-term kidney function (Figure 2A)

We examined the set of proteins with significant statistical differences between the 2 outcomes to verify the overarching trends. Pathway analysis via STRING^25^ of this 214-protein subset suggested enrichment of cellular metabolic processes that included cellular response to stress (n=26 proteins; FDR: 2x10E-3) and cell surface receptor signalling pathways (n=40; FDR: 3x10E-3) in SO versus GO, and enrichment of reactive oxygen species (ROS) detoxification (n=107 proteins; FDR: 2x10E-3) in GO versus SO . Among the predominant pathways (via KEGG^26^ database) were metabolic dysregulation (n=107, FDR 7.13x10E-6) and tight junction molecules (n=40 proteins; FDR 3x10E-3).

As a further assessment of the proteomic results, we performed orthogonal quantitative comparisons of selected key proteins by immunoblotting (Figure S1B, SDC, http://links.lww.com/TP/B599). In SO versus GO we were able to reproduce the observation of significantly increased expression of the apoptotic signal transduction protein STAT-1, observed in proteomics data with 7-fold SO/GO, p=0.03, and by immunoblot with p=0.02 (Figure 2B & Figure S2A, SDC, http://links.lww.com/TP/B599). The proteomics data also suggested a role for a profibrotic response, with TGF-β1 (observed in proteomics data with 7-fold SO/GO, p=0.034). We were unable to quantify TGF-β1 confidently due to technical noise, however immunoblotting of PDGFRα (which has a synergetic role in the onset and propagation of fibrosis) did indicate elevation (p=0.01) (Figure 2B & Figure S2B, SDC, http://links.lww.com/TP/B599). In GO versus SO we were able to observe enrichment of several notable antioxidant and cytoprotective proteins by western blot including, thioredoxin 1 (p=0.005), glutathione S transferase p=0.02) and peroxiredoxin-3 (p= 0.04) (Figure 2C & Figure S3A, B,C, SDC, http://links.lww.com/TP/B599). Interestingly, catalase was not observed to be enriched by either the proteomic analysis (p>0.05) or immunoblotting (p>0.05) (Figure S3D, SDC, http://links.lww.com/TP/B599)

**Discussion**

Assessment of donor organ quality using current clinical decision making frequently results in a conservative donor organ selection with the consequence that a large number of potentially transplantable organs are declined^8,9^. The limitations of the current clinical methods are highlighted in our study, wherein neither AKIN, KDPI nor Remuzzi classification predicted which donor kidneys in our cohort would have suboptimal function after transplantation, despite limiting assessment to 2 ‘extreme’ posttransplant outcome groups with minimal ambiguity.

In contrast, an exploratory proteomics study indicated significant differences in the proteomes of donor kidney biopsies from the 2 groups allowing clear separation by nonsupervised clustering. Furthermore, the bio-mechanistic differences most strongly suggested by the proteomics study (particularly a role for ROS-induced injury and recovery capability) were consistent with follow-up immunoblotting and are biologically plausible in the context of previously reported studies. As we and others have previously reported, brain death leads to dysregulation of metabolic pathways and mitochondrial dysfunction that results in ROS formation, causing cellular injury in the kidney^27^. The dynamic relationship between ROS and TGF-β has also been examined extensively in vitro and in vivo^28–30^. The synergistic action of TGF-β and PDGFRα can promote the differentiation of pericytes and the recruitment of fibroblasts and myofibroblasts, causing irreversible changes to the extracellular matrix, promoting fibrosis and damage to renal tubules^28,31–34^ and PDGFRα also contributes to kidney fibrosis through a maladaptive process of wound healing^35–37^. Furthermore, in many models of fibrosis and atherosclerosis, activation of PDGF receptors leads to STAT-1 phosphorylation in a JAK1/2 dependent manner^38–41^. Fibrosis has been closely linked to deterioration of allograft function after transplantation^42^ . The antioxidant and cytoprotective proteins found to be elevated in GO kidneys would also be consistent with ROS-induced fibrosis playing a role in posttransplant kidney dysfunction. Antioxidant cellular mechanisms are generally activated after exposure to ROS mediated stress to reinstate a healthy cellular environment^43–45^, and the proteomic data and immunoblotting data are consistent with an elevated response in these pathways being associated with better posttransplant outcome.

Our pilot study results indicate that the current assessment methods of donor kidney quality may be augmented by proteomic profiles that indicate how the balance between injury and cytoprotection is disrupted and their association with long-term allograft function. Now that feasibility has been established, future work to identify a predictive protein profile can be performed in a much larger cohort with a broader continuum of outcomes.**ACKNOWLEDGMENTS**

We thank the UK QUOD consortium for the clinical samples analysed in this study. We are indebted to Dr Sergei Maslau for his support on the statistical analysis, Dr Roman Fischer for his expert input on mass spectrometry and Dr Letizia Lo Faro for her general advice and expertise.

**REFERENCES**

1. NHS Blood and Transplant. ANNUAL REPORT ON LIVING DONOR KIDNEY TRANSPLANTATION. Report for 2016/2017. https://nhsbtdbe.blob.core.windows.net/umbraco-assets-corp/5706/annual-report-on-living-donor-kidney-transplantation-2016_17.pdf. Published October 2017.

2. Tanriover B, Mohan S, Cohen DJ, et al. Kidneys at higher risk of discard: expanding the role of dual kidney transplantation. *Am J Transplant*. 2014;14:404-415.

3. Collins MG, Chang SH, Russ GR, McDonald SP. Outcomes of transplantation using kidneys from donors meeting expanded criteria in Australia and New Zealand, 1991 to 2005. *Transplantation*. 2009;87:1201-1209.

4. Singh RP, Farney AC, Rogers J, et al. Kidney transplantation from donation after cardiac death donors: lack of impact of delayed graft function on post-transplant outcomes. *Clin Transplant*. 2011;25:255-264.

5. NHS Blood and Transplant. Organ Donation and Transplantation Activity Report 2014/15. https://nhsbtmediaservices.blob.core.windows.net/organ-donation-assets/pdfs/activity_report_2014_15.pdf. Published 2015.

6. Moers C, Leuvenink HGD, Ploeg RJ. Non-heart beating organ donation: overview and future perspectives. *Transpl Int*. 2007;20:567-575.

7. Metzger RA, Delmonico FL, Feng S, Port FK, Wynn JJ, Merion RM. Expanded criteria donors for kidney transplantation. *Am J Transplant*. 2003;3 Suppl 4:114-125.

8. Mittal S, Adamusiak A, Horsfield C, et al. A Re-Evaluation of Discarded Deceased Donor Kidneys in the UK: Are Usable Organs Still Being Discarded? *Transplantation*. 2017;101:1698-1703.

9. Callaghan CJ, Harper SJF, Saeb-Parsy K, et al. The discard of deceased donor kidneys in the UK. *Clin Transplant*. 2014;28:345-353.

10. Nuffield Department of Surgical Sciences. Medical Sciences Division. https://www.nds.ox.ac.uk/research/quod.

11. Sigdel TK, Ng YW, Lee S, et al. Perturbations in the urinary exosome in transplant rejection. *Front Med*. 2014;1:57.

12. Sigdel TK, Gao Y, He J, et al. Mining the human urine proteome for monitoring renal transplant injury. *Kidney Int*. 2016;89:1244-1252.

13. Sigdel TK, Lee S, Sarwal MM. Profiling the proteome in renal transplantation. *Proteomics Clin Appl*. 2011;5:269-280.

14. Sigdel TK, Salomonis N, Nicora CD, et al. The identification of novel potential injury mechanisms and candidate biomarkers in renal allograft rejection by quantitative proteomics. *Mol Cell Proteomics*. 2014;13:621-631.

15. Levey AS, Bosch JP, Lewis JB, Greene T, Rogers N, Roth D. A more accurate method to estimate glomerular filtration rate from serum creatinine: a new prediction equation. Modification of Diet in Renal Disease Study Group. *Ann Intern Med*. 1999;130:461-470.

***References 16-45 can be found in Material and Methods, SDC, http://links.lww.com/TP/B599; References section***

**Table 1**. Donor and recipient demographic and clinical characteristics associated with the analysed kidney samples

**Suboptimal Outcome (n=19) Good Outcome (n=19) *P* Value**

**Donor characteristics**

Age (yr)* 56 + 13 54 + 11 0.5

Gender (%)

Male 10 (53) 7 (37) 0.5

Race (%)

White 18 (95) 16 (84) 0.6

Other 1 (5) 3 (16)

Weight (Kg)* 85 + 15 80 + 17 0.3

Height (cm)* 170 + 9 167 + 9 0.4

S-Cr terminal (mg/dl)*^/^^ 1.3 + 1 0.8 + 0.4 0.1

Cause of death (%) 0.6

Intracranial haemorrhage 10 (53) 10 (53)

Hypoxic brain injury 4 (21) 2 (10)

Other 5 (26) 7 (37)

**AKIN classification (%)** 0.5

No AKIN 16 (85) 15 (79)

1 1 (5) 3 (16)

2 1 (5) 1 (5)

3 1 (5) 0

**Recipient characteristics**

Age (yr)* 50.1 + 13.5 48.4 + 13.6 0.5

Gender (%)

Male 13 (68) 13 (68) 1

Race (%)

White 15 (79) 10 (53) 0.17

Other 4 (21) 9 (47)

HLA mismatches (%) 0.22

1 3 (16) 3 (16)

2 5 (26) 7 (37)

3 11 (58) 7 (37)

4 0 2 (10)

CIT (h) 1

0- 12 6 (31) 6 (31)

12- 26 13 (69) 13 (69)

**Posttransplantation Kidney**

**function (mean eGFR ml/min/1.73m^2^ )*** <0.0001

3-month 29.8 + 7 65.1 + 8

12-month 35.9 + 6 73.0 +18

**Means +/- SD values are reported, ^ Correlation of SCr and 12-month eGFR shown in Figure S4, SDC, http://links.lww.com/TP/B599*

**FIGURE LEGENDS**

**Figure 1. *Clinical assessment of donor kidney biopsies by KDPI & Remuzzi scoring and proteomic profiling.***

**1A.** KDPI and Remuzzi scoring failed to discriminate the donor kidneys in risk of suboptimal transplantation outcomes. KDPI scoring was performed using the KDPI online calculator; there was not significant difference between the KDPI scoring of the donors included in the study (*Mann Witney test; p=0.69*; Data show Means+/-SD).

**1B**. Histological analysis shows there was not significant difference between the Remuzzi scoring of the donors included in the study (*Mann Witney test; p=0.1*; Data show Means+/-SD)

**1C.** **Proteomic profiling of donor kidneys.**

Kidney tissue proteome profiles discriminate donors by unsupervised principle component analysis on the basis of allograft function after transplantation.

**Figure 2.** **Dysreg*ulated protein classes in kidney tissue between donors with suboptimal and good transplantation outcomes.***

***2A*.** Supervised HCA segregated individual donor kidneys in 2 distinct groups according to 3-month kidney function posttransplantation. S1, S2, S3, S4, S5: Individual donor kidneys with suboptimal outcomes. G1, G2, G3, G4, G5: Individual donor kidneys with good outcome. Association between the HCA derived dendrogram from donor kidney biopsies analysis and the recipient kidney function recorded at 3-month (used for selection) and 12-month posttransplantation (retrospective). GF: graft failure, MV: missing value.

**2B.** ***STAT-1 and PDGFRα are enriched in donor kidneys with SO****.*

Western blot analysis of STAT-1 and PDGF Rα on the rest of the selected sample cohort of n= 28 biopsy samples (n=14 suboptimal and n=14 good outcome cohort). Normalised by ß-actin, densitometry analysis shows significant increased levels of STAT-1 and PDGFRα in SO associated donor kidney biopises (p≤0.05; Data show Means+/-SD .

**2C.** ***Cytoprotective proteins are enriched in the donor kidneys with GO*.**

Western blot analysis of GST, PRX3 and TRX1 on the rest of the selected sample cohort n=28 DBD kidney biopsies (n=14 suboptimal and n=14 good outcome cohort). Normalised by ß-actin densidometric analysis shows significant increased levels of TRX1, GST, and PRX3 in GO associated donor kidney biopises (p≤0.05; Data show Means+/-SD).
