# Supplementary material for: Subclinical Changes in Deceased Donor Kidney Proteomes Are Associated With 12-month Allograft Function Posttransplantation—A Preliminary Study
Source: Transplantation. 2019 Jan 28;103(2):323–8. doi: 10.1097/TP.0000000000002358 (PMC6365243; doi:10.1097/TP.0000000000002358)
Supplement: SUPPLEMENTARY MATERIAL [file tp-103-323-s002.pdf]

SDC, Materials and Methods

Immunoblotting

Selected proteins implicated by proteomic analysis of the 10-sample subset were analysed in the remaining 28 samples by immunoblotting. Samples containing 15 µg of protein were denatured at 95°C for 5 minutes in Laemmli buffer and loaded into 8–12% precast SDSPAGE gels (Bio-Rad, USA). Proteins separated by SDS-PAGE were transferred to hydrophobic PVDF membranes (Merck Millipore, USA) in transfer buffer (25 mM Tris, 192 mM glycine and 10% methanol) overnight. PVDF membranes were blocked for 1 hour in TBST buffer (25 mM Tris, pH 7.5, 0.15 M NaCl, 0.05% Tween 20) containing 5% milk. Membranes were incubated for overnight at 4 °C with anti TRX1 (ab26320, 1:1000), PRX3 (ab73349, 1:1000), Catalase (ab16731, 1:1000), GST (ab53940, 1:3000) (Abcam, UK), STAT-1 (mab14901, 1:1000), PDGFRα (af307na, 1:800) (R&D Systems, US). Rabbit monoclonal anti Beta-actin served as a loading control (ab8227, 1:5000) (Abcam, UK). Membranes were washed for 30 minutes with 5 changes of wash buffer and then incubated at room temperature for 1 hour in blocking buffer containing a 1:5000 dilution of Dye-800-conjugated anti-mouse, rabbit, or -goat secondary antibody (Li-Cor, Nebraska, USA), and visualization was performed with Odyssey CLx (Li-Cor Nebraska, USA). Detected signal was quantified and normalized to β-actin on the same blot.

Table S1: Remuzzi scoring

	Suboptimal Outcome (n=19)	Good Outcome (n=19)	*P* Value
**Remuzzi score (%)**			0.1
0- 3	13 (68)	15 (79)	
4-8	1 (5)	1 (5)	

Figure S1

A. Sample selection diagram

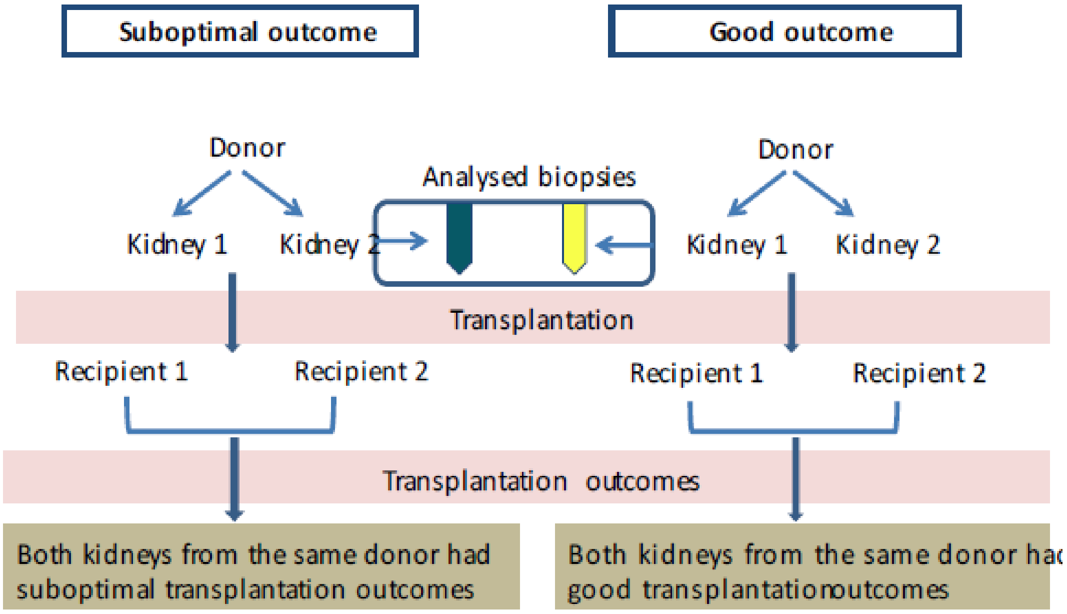

B. Experimental workflow

I. LFQ MS/MS Proteomic analysis workflow

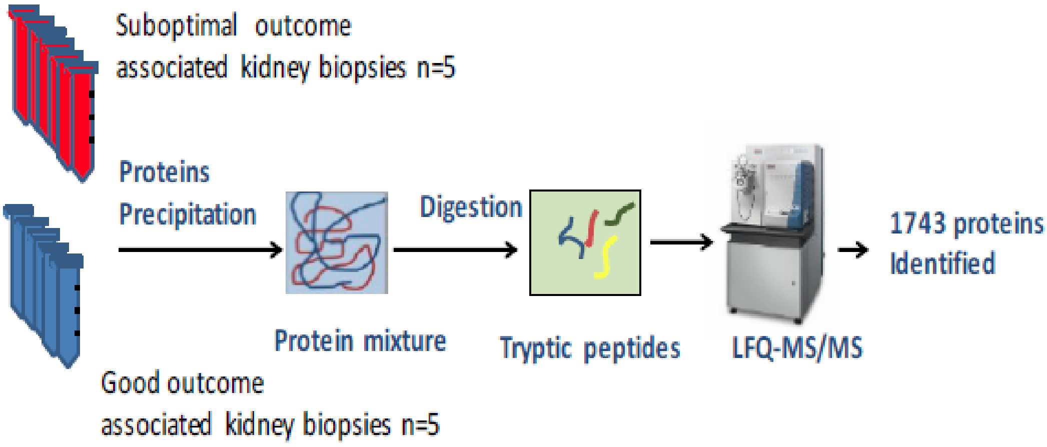

II. Immunoblotting analysis

Figure S1. ***Experimental design***

1A. The kidney biopsy sample selection diagram shows the selection of donors included in the study. Selected donors offered both kidneys as single transplants with both allografts giving rise to similar outcome suboptimal or good allogarfat function. GO donor kidneys had a mean 3-month eGFR= 65.2 +/- 8 ml/min/1.73 m^2 (25th and 75th percentiles for eGFR were 62 and 70.25 ml/min/1.73 m^2 respectively). SO donor kidneys had a mean 3-month eGFR= 29.8+/-7ml/min/1.73 m^2 (25th and 75th percentiles for eGFR were 37.2 and 24.8 ml/min/1.73 m^2 respectively). The difference of the posttransplant 3-month eGFR values between the 2 cohorts (SO vs GO) was significant different (P<0.0001; Mann-Whitney test).

1B. The experimental workflow contained 2 stages. (1) Initially, n=10 individual kidney biopsies (n=5 samples per transplantation outcome) were analysed by label-free quantitative MS. Biopsy sample homogenates were precipitated, proteins were enzymatically digested to tryptic peptides and analysed by tandem mass spectrometry. Bioinformatic analysis resulted in the identification of 1743 proteins (FDR<1%). (2). Western blot analysis was performed on the rest of the selected cohort of DBD kidney biopsies n=28 (n=14 biopsy samples per transplantation outcome). SO: suboptimal outcome, GO: good outcome.

Figure S2

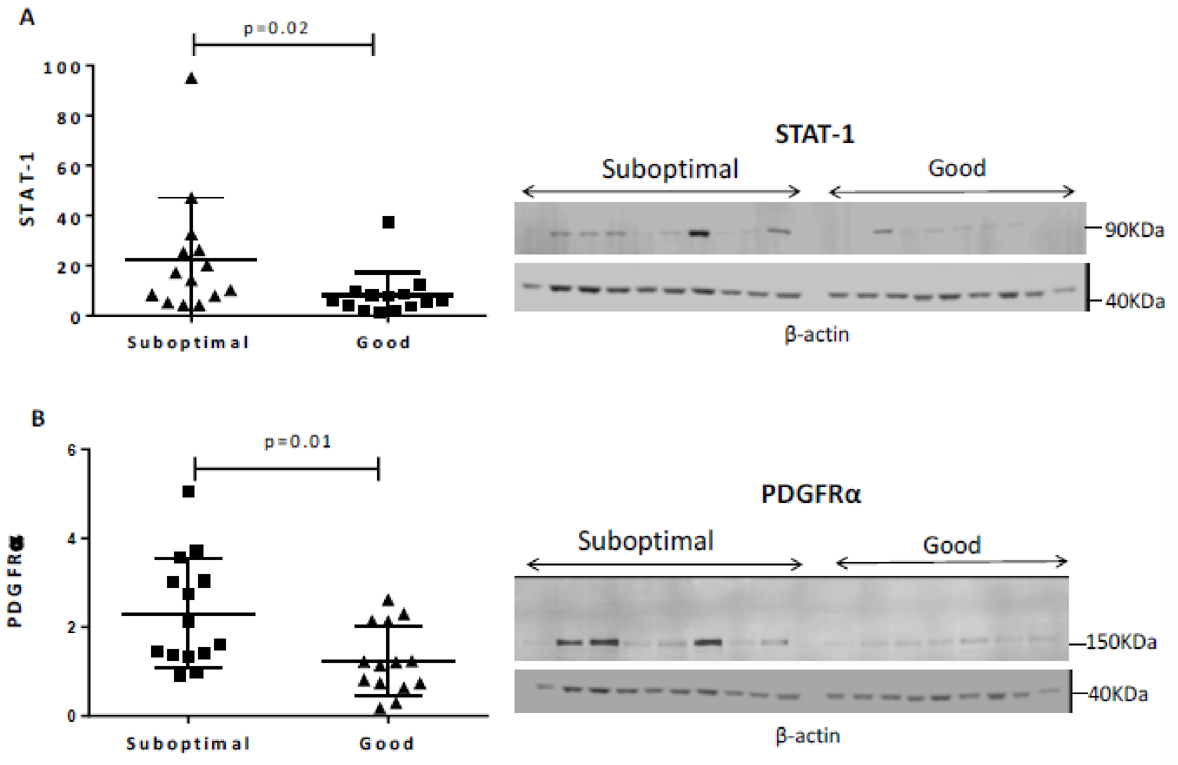

Figure S2. ***STAT-1 and PDGFRα are enriched in donor kidneys with SO***.

Western blot analysis of STAT-1 (**A**) and PDGF Rα (**B**) on the rest of the selected cohort of n= 28 biopsy samples (n=14 suboptimal and n=14 good outcome cohort). Normalised by β-actin, densitometry analysis shows significant increased levels of STAT-1 and PDGFRα in SO associated donor kidney biopises ($p \leq 0.05$).

Figure S3

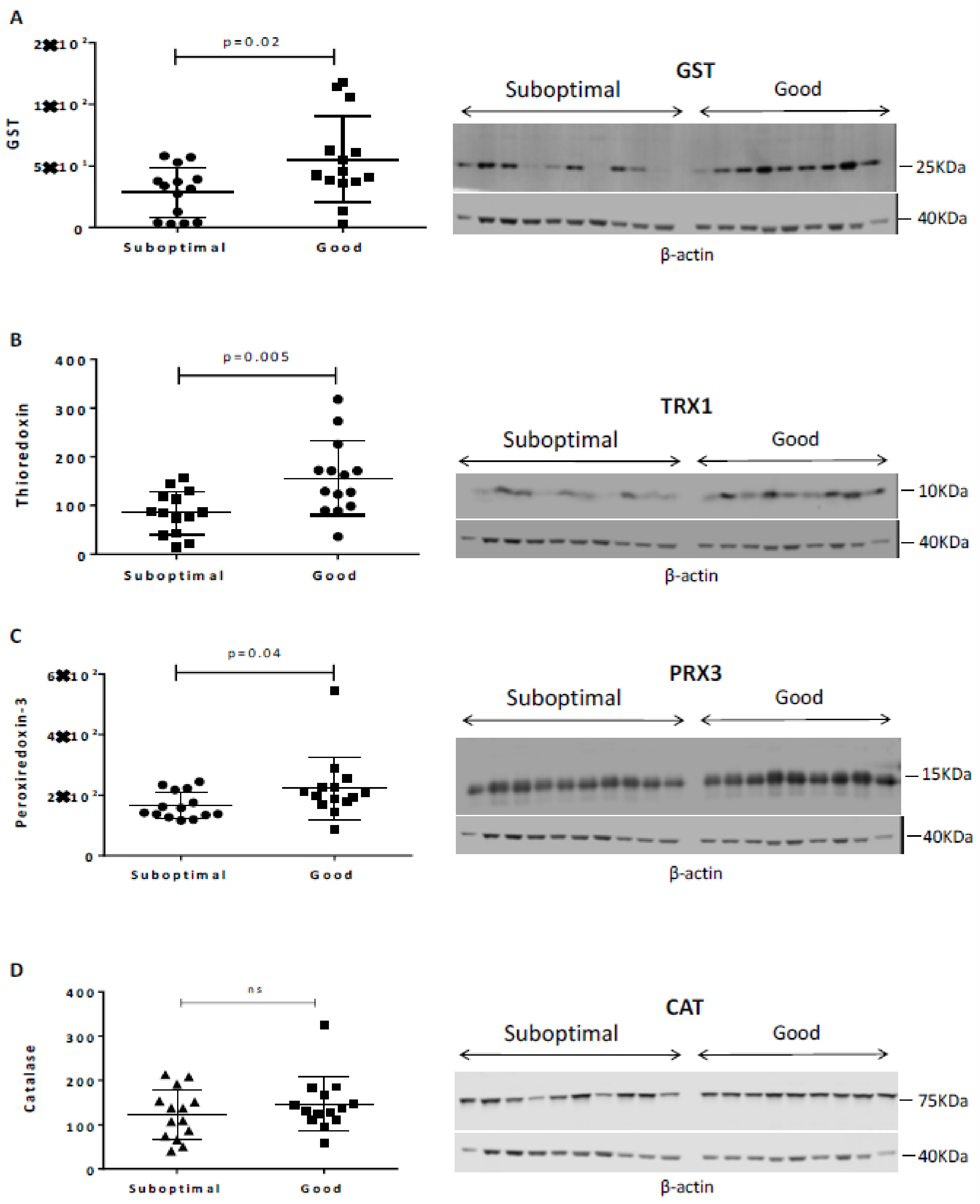

Figure S3. ***Antioxidant proteins are enriched in the donor kidneys with GO*.**

Western blot analysis of GST (**A**), TRX1 (**B**), PRX3 (**C**) and CAT (**D**) on the rest of the selected cohort of n=28 DBD kidney biopsies (n=14 suboptimal and n=14 good outcome cohort).

Normalised by β-actin densidometric analysis shows significant increased levels of TRX1, GST, and PRX3 in GO associated donor kidney biopises ($p \leq 0.05$). CAT was not significantly altered.

Figure S4

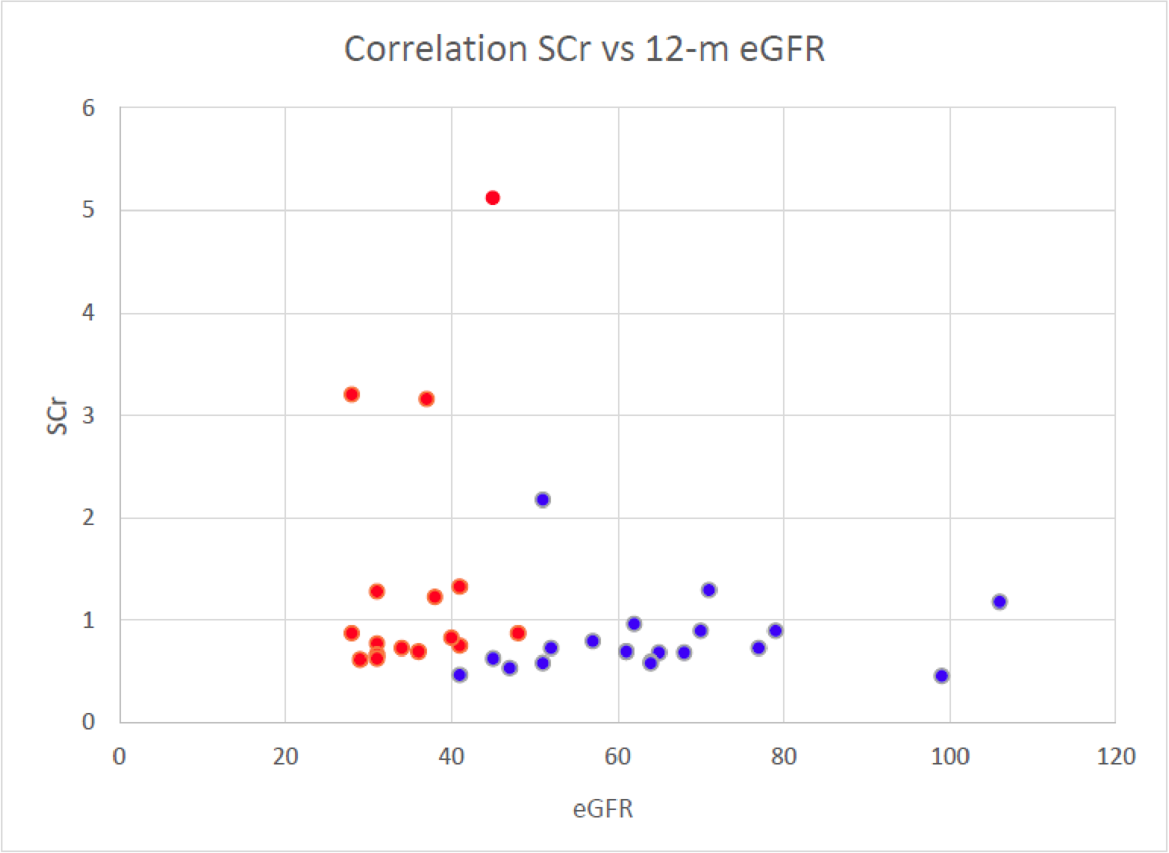

Figure S4. ***Correlation of Serum Creatinine versus 12-month eGFR***

A (nonstratified) comparison between donor serum creatinine and 12-mo eGFR did not find a strong correlation (Spearman Rho=-0.007; p<=0.65); plotting the data reveals that the observed mean difference is likely due to a few anomalous readings.

References

16. Gandolfini I, Buzio C, Zanelli P, et al. The kidney donor profile index (KDPI) of marginal donors allocated by standardized pretransplant donor biopsy assessment: Distribution and association with graft outcomes. *Am J Transplant*. 2014;14:2515-2525.
17. Ricci Z, Cruz DN, Ronco C. Classification and staging of acute kidney injury: beyond the RIFLE and AKIN criteria. *Nat Rev Nephrol*. 2011;7:201-208.
18. Edward Sharplesa, Anna Casulab CB. UK Renal Registry 19th Annual Report: Chapter 3 Demographic and Biochemistry Profile of Kidney Transplant Recipients in the UK in 2015: National and Centre-specific Analyses. *Nephron*. 2016;132:99-110.
19. Hilbrands LB, Wetzels JFM, Remuzzi G, et al. Long-term outcome of renal transplantation from older donors. *N Engl J Med*. 2006;354:343-352.
20. Levin Y. The role of statistical power analysis in quantitative proteomics. *Proteomics*. 2011;11:2565-2567.
21. Fischer R, Kessler BM. Gel-aided sample preparation (GASP)--a simplified method for gel-assisted proteomic sample generation from protein extracts and intact cells. *Proteomics*. 2015;15:1224-1229.
22. Trudgian DC, Thomas B, McGowan SJ, Kessler BM, Salek M, Acuto O. CPFP: A central proteomics facilities pipeline. *Bioinformatics*. 2010;26:1131-1132.
23. Trudgian DC, Ridlova G, Fischer R, et al. Comparative evaluation of label-free SINQ normalized spectral index quantitation in the central proteomics facilities pipeline. *Proteomics*. 2011;11:2790-2797.
24. Caraux G, Pinloche S. PermutMatrix: a graphical environment to arrange gene expression profiles in optimal linear order. *Bioinformatics*. 2005;21:1280-1281.
25. Szklarczyk D, Morris JH, Cook H, et al. The STRING database in 2017: qualitycontrolled protein–protein association networks, made broadly accessible. *Nucleic Acids Res*. 2017;45(D1):D362-D368.
26. Kanehisa M, Sato Y, Kawashima M, Furumichi M, Tanabe M. KEGG as a reference resource for gene and protein annotation. *Nucleic Acids Res*. 2016;44(D1):D457-62.
27. Akhtar MZ, Huang H, Kaisar M, et al. Using an integrated -omics approach to identify key cellular processes that are disturbed in the kidney following brain death. *Am J Transplant*. 2016;16:1421-1440.

28. Liu R-M, Desai LP. Reciprocal regulation of TGF-β and reactive oxygen species: A perverse cycle for fibrosis. *Redox Biol*. 2015;6:565-577.

29. Djamali A, Vidyasagar A, Adulla M, Hullett D, Reese S. Nox-2 is a modulator of fibrogenesis in kidney allografts. *Am J Transplant*. 2009;9:74-82.

30. Jenkins RH, Martin J, Phillips AO, Bowen T, Fraser DJ. Transforming growth factor β1 represses proximal tubular cell microRNA-192 expression through decreased hepatocyte nuclear factor DNA binding. *Biochem J*. 2012;443:407-416.

31. Eddy AA. Overview of the cellular and molecular basis of kidney fibrosis. *Kidney Int Suppl*. 2014;4:2-8.

32. Zhong X, Chung ACK, Chen H-Y, Meng X-M, Lan HY. Smad3-mediated upregulation of miR-21 promotes renal fibrosis. *J Am Soc Nephrol*. 2011;22:1668-1681.

33. Richter K, Konzack A, Pihlajaniemi T, Heljasvaara R, Kietzmann T. Redox-fibrosis: Impact of TGFβ1 on ROS generators, mediators and functional consequences. *Redox Biol*. 2015;6:344-352.

34. Daehn I, Casalena G, Zhang T, et al. Endothelial mitochondrial oxidative stress determines podocyte depletion in segmental glomerulosclerosis. *J Clin Invest*. 2014;124:1608-1621.

35. Boor P, Ostendorf T, Floege J. PDGF and the progression of renal disease. *Nephrol Dial Transplant*. 2014;29 Suppl 1:i45-i54.

36. Eitner F, Ostendorf T, Kretzler M, et al. PDGF-C expression in the developing and normal adult human kidney and in glomerular diseases. *J Am Soc Nephrol*. 2003;14:1145-1153.

37. Floege J, Eitner F, Alpers CE. A new look at platelet-derived growth factor in renal disease. *J Am Soc Nephrol*. 2008;19:12-23.

38. Vignais ML, Sadowski HB, Watling D, Rogers NC, Gilman M. Platelet-derived growth factor induces phosphorylation of multiple JAK family kinases and STAT proteins. *Mol Cell Biol*. 1996;16:1759-1769.

39. He C, Medley SC, Hu T, et al. PDGFRβ signalling regulates local inflammation and synergizes with hypercholesterolaemia to promote atherosclerosis. *Nat Commun*. 2015;6:7770.

40. Paukku K, Valgeirsdóttir S, Saharinen P, Bergman M, Heldin CH, Silvennoinen O. Platelet-derived growth factor (PDGF)-induced activation of signal transducer and

activator of transcription (Stat) 5 is mediated by PDGF beta-receptor and is not dependent on c-src, fyn, jak1 or jak2 kinases. *Biochem J*. 2000;345 Pt 3:759-766.

41. Choudhury GG, Marra F, Kiyomoto H, Abboud HE. PDGF stimulates tyrosine phosphorylation of JAK 1 protein tyrosine kinase in human mesangial cells. *Kidney Int*. 1996;49:19-25.

42. O'Connell PJ, Zhang W, Menon MC, et al. Biopsy transcriptome expression profiling to identify kidney transplants at risk of chronic injury: a multicentre, prospective study. *Lancet (London, England)*. 2016;388:983-993.

43. Chouchani ET, Pell VR, Gaude E, et al. Ischaemic accumulation of succinate controls reperfusion injury through mitochondrial ROS. *Nature*. 2014;515:431-435.

44. Gorrini C, Harris IS, Mak TW. Modulation of oxidative stress as an anticancer strategy. *Nat Rev Drug Discov*. 2013;12:931-947.

45. Eltzschig HK, Eckle T. Ischemia and reperfusion—from mechanism to translation. *Nat Med*. 2011;17:1391-1401.
